# Radiation Oncology Workforce Recruitment Survey of 2000–2010 Graduates: Is There a Need for Better Physician Resource Planning?

**Published:** 2012-03-31

**Authors:** Shaun Loewen, Michael Brundage, Keith Tankel, Alysa Fairchild, Theresa Trotter, Ericka Wiebe, Paris Ann Ingledew, Teri Stuckless, Don Yee

**Affiliations:** 1 Dept. of Radiation Oncology, Cross Cancer Institute, Edmonton, Alberta, Canada; 2 Dept. of Radiation Oncology, Cancer Centre of Southeastern Ontario, Kingston, Ontario, Canada; 3 Dept. of Radiation Oncology, Tom Baker Cancer Centre, Calgary, Alberta, Canada; 4 Dept. of Radiation Oncology, Sunnybrook Health Sciences Centre, Toronto, Ontario, Canada; 5 Dept. of Radiation Oncology, Fraser Valley Cancer Centre, Surrey, British Columbia, Canada; 6 Dept. of Radiation Oncology, Dr. H. Bliss Murphy Cancer Centre, St. John’s, Newfoundland, Canada

## Abstract

**Purpose of the Study:**

To survey employment and training characteristics of Canadian radiation oncology training program graduates and foreign medical graduates with Canadian radiation oncology post-graduate education or specialist certification.

**Methods:**

A 38-question, web-based survey was distributed to radiation oncologists who completed specialty training between 2000–2010.

**Results:**

Out of 256 radiation oncologists contacted, 148 completed the survey (58% response rate). Thirty-two respondents (22%) were foreign MD graduates. One-hundred and fifteen respondents (78%) undertook fellowship training after residency. Many Canadian MD graduates (77%) and foreign MD graduates (34%) had staff positions in Canada, while 11% of all respondents had staff positions outside Canada, and 21% did not have a commitment for staff employment. Of the 31 respondents without a staff position, 22 graduated from Canadian residency training in 2009 or 2010, and 21 had completed medical school training in Canada.

**Conclusions:**

The majority of respondents were successful in securing staff positions in Canada. A sizeable proportion extended training with fellowships. New graduates may have more difficulty in finding Canadian staff positions in radiation oncology in the near future. Implications for specialty training programs and for an improved national strategy for physician resource planning are discussed.

## Introduction

Co-ordinated manpower planning of radiation oncology services in Canada has had a troubled history. During the 1980s and 1990s, shortages of radiation oncologists contributed to unacceptably high service workloads and prolonged wait times for radiotherapy, prompting some Canadian provinces to send patients to the United States for radiation treatments.^1^,^2^ Despite successful efforts to attract Canadian medical students to radiation oncology training programs to meet the staffing shortfall, graduates were met with few job opportunities due to inadequate and untimely local funding for cancer centre expansion and physician recruitment.^3^,^4^ Lack of job availability resulted in a sharp decline of the number of Canadian radiation oncology residents in the late 1990s due to cuts in trainee positions, residents leaving the specialty prior to completing training, and declining medical student interest in the specialty.^5^

Public funding for radiation oncologist positions increased in 2000, and for those residents who remained in training, career opportunities were abundant and exceeded graduate supply. A significant number of unfilled staff positions prompted a published statement in 2001 from the Canadian Association of Radiation Oncology (CARO) to inform medical students that career opportunities in radiation oncology were plentiful with a promising outlook.^6^ To meet immediate staffing needs, active recruitment of foreign radiation oncologists was endorsed by CARO and the Royal College of Physicians and Surgeons of Canada (RCPSC).^6^ Residency programs expanded and the specialty’s popularity as a career choice with medical students soared.^5^

There were 201 radiation oncology residents and fellows in Canadian training programs in 2010–2011,^5^ equivalent to nearly half of the estimated 437 staff radiation oncologists working in Canada today.^7^ The 2009 CARO Workforce Survey predicted a surplus of more than 30 graduates by 2012,^8^ and has again raised fears of job availability for new graduates. Furthermore, a number of reports have been published that have identified recent graduates from a variety of specialties, including radiation oncology, who are having difficulties in finding work.^9^–^12^ The RCPSC has launched a larger in-depth study of the issue to determine contributing factors to physician oversupply.^13^

Currently, little is known about the employment trends and characteristics of the Canadian radiation oncology workforce. The rationale for this study was to collect data that would better inform national manpower planning processes, and better inform the curriculum of current trainees, such that perceived opportunities for enhancing training cited by graduates could be addressed effectively. Our primary objective was to document success rates of Canadian-trained radiation oncologists in finding staff positions over the past decade and to identify employment trends. Secondary objectives were to gain insight and perspective on training characteristics, recruitment experiences, employment preferences, and perceptions of manpower planning. Our data provides real-world outcomes of workforce recruitment with market forces, and independent from radiation oncology manpower requirements and workforce modeling. Survey participants also included foreign MD graduates with Canadian training and/or certified Fellows of the Royal College of Physicians of Canada (FRCPC) in radiation oncology to capture data from the entire potential recruitment supply to the Canadian radiation oncology workforce.

## Methods

Residency program directors and fellowship directors from 13 Canadian radiation oncology residency programs were contacted to identify Canadian and foreign MD graduates from their program and obtain email contact information. Additional online membership databases from CARO, RCPSC, and provincial medical colleges were used to supplement participants and cross-reference email contact information. Participant eligibility was restricted to radiation oncologists who received Canadian residency training and/or Canadian specialist certification between 2000 and 2010, or in the case of international MD graduates in fellowship training, enrolled in an accredited Canadian training program for the 2009–2010 academic cycle. The study period was limited to the past 10 years as this study was not intended to provide a complete historical review of the specialty but rather an assessment of recent trends.

The survey was conducted using principles from the Total Design Method of conducting mail surveys.^14^ Each eligible participant was sent a secure explanatory e-mail containing a link to access the survey on the proprietary website *Surveymonkey.com*. The first webpage provided the rationale for the survey and required participants to click a check box to continue, indicating informed consent. The survey contained 38 multiple-choice and open-ended questions about training demographics, employment characteristics and recruitment experiences, and opinions on workforce issues within the specialty, employment preferences, and manpower planning in Canada. Participants had the option to skip questions and missing responses were categorized as ‘unknown’. Mean response rate per question was 98%. Two electronic reminders were sent at approximately 2-week intervals to non-responders after the initial invitation. Invitations to participate were sent on June 15^th^, 2010 and survey responses were accepted until July 25^th^, 2010.

### Statistical Analysis

Anonymized, aggregate responses from returned surveys were analyzed using an Excel database and the survey website tools. Descriptive statistics were used to summarize the data and describe responses. Frequency and percentages were used to summarize the categorical responses. Observed sample size accuracy was calculated with 95% confidence level and 50% categorical response, resulting in a confidence interval of 5%. Chi-square tests were used to compare demographics of actual respondents vs. eligible respondents, and were also used to compare employment status of respondents by gender, radiation oncology training location, MD degree origin, and residency graduation. Similar distribution analyses were performed for respondents with fellowship training by residency graduation. Fisher’s exact test was used when the cell frequency was less than 5. A *p*-value of ≤0.05 was used for statistical significance.

## Results

### Respondents’ demographics

Canadian radiation oncology training programs and databases provided by CARO, RCPSC, and the provincial medical colleges identified 269 participants. Email invites to 13 were undeliverable, leaving 256 eligible respondents. Of these, 148 participated for a survey response rate of 58% (148/256), and represent 55% of identified participants.

Demographic features of respondents vs. eligible respondents according to gender, residency training location, and graduating year were similar with non-significant *p*-values between 0.37–0.79 (Table 1). A majority of respondents completed radiation oncology residency training before the age of 34 and had a Canadian medical degree. Over 87% of respondents completed their radiation oncology residency training in Canada, most commonly in Ontario. Ten respondents had additional medical certification in family medicine or internal medicine. Of the respondents, 27% held Master’s or PhD degrees.

### Fellowship training in radiation oncology

A majority of respondents (78%) pursued fellowship after residency training, 76% completing at least 12 months of fellowship training after radiation oncology residency, and 24% completing more than 12 months (Table 2). When separated by graduation year, 63% (26/41) of respondents who graduated before 2006 undertook fellowships compared to 80% (82/102) of respondents who graduated in 2006 or later (*p* = 0.023). There were more 2006–2010 graduates who completed >12 months of fellowship training (22/28) and completed, or intended to complete, more than one fellowship (10/15), but the proportion of graduates completing >12 months of fellowship or multiple fellowships has not changed significantly over the past decade (*p =* 0.73 and 0.35, respectively). Ontario, the United States, and Europe were the top 3 locations for fellowships. Most respondents included radiotherapy delivery technology and techniques and/or cancer site-specific clinical training as the major focus of their fellowship. The amount of research focus varied, but purely clinical fellowships were rare. Thirteen respondents used fellowship opportunities to perform post-graduate work leading to a Master’s or PhD degree.

Reasons to undertake fellowship training were: to be more competitive in the job market, to gain specific clinical experience, and to pursue research interests. In contrast, reasons for not pursuing a fellowship were: the availability of staff positions where fellowship training was not required, or preference to enter the workforce immediately after residency.

### Respondents’ employment experiences and characteristics

Most respondents (79%) had secured a staff position, the majority of which are in Canada (Table 3). Eighty-nine of 115 respondents with Canadian medical degrees and 11/32 with foreign medical degrees have staff positions in Canada, while 11/32 respondents with foreign medical degrees and 5/115 with Canadian medical degrees have staff positions outside Canada. The proportion of male vs. female respondents employed in Canada was similar (*p* = 0.45). However, statistical differences were seen when comparing employment status of respondents with Canadian MDs vs. foreign MDs (*p* <0.0001) and Canadian vs. non-Canadian residency training (*p* <0.0001). The largest difference in staff employment status was seen to be a function of radiation oncology residency graduation era, i.e. 2000–2008 vs. 2009–2010 (*p* <0.0001).

Respondents were more likely to obtain an employment offer for a staff position prior to completion of fellowship (87%) than prior to completion of residency (47%), and once employed, most respondents worked full-time (87%) (Table 4). Most respondents who undertook fellowships (90%) continued to use the experience gained in fellowship training in their current practice. Few respondents (17/117) reported relocating after initial staff employment and fewer (4/117) experienced periods of unemployment after initial employment. A significant number of respondents (110/117) incorporated research activities as a part of their practice once a staff position was obtained. Research funding was available for 59% (69/117) of respondents and 49% (56/114) had protected time for research.

### Job supply and career plans

Respondents’ opinions on their recruitment experiences and career plans are summarized in Table 5. Most respondents felt that there were insufficient resources available to help find staff employment in Canada. Of the 30 additional comments related to this question, the CARO website was cited as the primary resource for finding radiation oncology positions in Canada. Several respondents indicated that some employment opportunities were not readily publicized and instead relied on personal communications or networking for distribution to potential job seekers. After residency and/or fellowship training, 27% (36/135) of respondents experienced difficulty in finding staff employment in Canada and 83% of respondents intended to stay in Canada after training (121/145). Twenty-four out of 145 respondents (17%) intended to leave Canada and were mostly foreign MD graduates (17/24).

More than half of the respondents preferred to work in a large or medium-sized academic cancer centre, and approximately one-quarter were willing to work in a medium-sized regional cancer centre with more than 5 radiation oncologists. Only 11% of respondents were willing to work in a small regional cancer centre with fewer than 5 radiation oncologists.

With regards to respondents’ attitudes towards job supply, approximately 50% of respondents felt that there were not enough positions in Canada to meet the potential graduate supply at the time when respondents completed their residency and/or fellowship training and more respondents (76%) thought that issues of employment were worse for current graduating residents and fellows. Most respondents (88%) thought that post-residency fellowship training improved their chances of securing a position at an academic cancer centre. About half of respondents thought that fellowship training encouraged hiring at regional cancer centres, 28% were neutral, and 15% thought that there was no added benefit of fellowship training.

## Discussion

This survey represents the first formal career initiation assessment of the Canadian radiation oncology workforce. It provides objective data regarding workforce demographics and includes training and employment patterns of radiation oncologists from Canadian training programs. It illustrates significant concerns radiation oncologists have about staff employment opportunities in Canada following graduation and emphasizes the need for a co-ordinated national strategy in the management of physician resources.

Our respondent population provides adequate representation of radiation oncology graduates from 2000–2010 without undue bias from gender, graduating year, and training location (non-significant *p*-values in distribution analyses). Our data represent over a decade’s worth of workforce recruitment and assesses approximately one-quarter of the existing radiation oncology staff workforce in Canada. A recognized limitation of our study is the self-reporting nature of the survey method as it may incorporate responder or selection bias. As with all surveys, results are based on opinions and self-assessment. Consequently, issues with poor recall, misunderstanding of questions, and intentional deception may contribute to inaccuracies in the data.

Foreign MD graduates were included in our survey to sample opinions and career plans from graduates other than those who trained in Canadian medical schools. Although Canadian training programs provided contact information of foreign MD graduates with residency training in Canada, the true number of foreign graduates with Canadian fellowship training and/or licensed to practice in Canada from 2000–2010 is unknown due to lack of up-to-date contact information in personnel databases or loss of RCPSC member registration due to non-payment. Therefore, we recognize that our data may not be representative of employment characteristics, Canadian recruitment and retention, and migration rates of all foreign MD graduates over the study period.

The majority of respondents were trained at Canadian medical schools. The relatively low number of survey participants who graduated in 2000–2005 is proportional to information provided by residency training programs across Canada, when at its lowest point in 2003, there were 7 graduates nationally. The number of 2006–2010 graduates from Canadian training programs rose sharply to an average of 32 per year as training programs re-expanded after the mass exodus of trainees from the late 90s job shortage. In turn, this has dramatically increased the supply of graduates seeking staff positions. Our survey confirmed that a significantly higher proportion of 2006–2010 graduates, compared to 2000–2005 graduates, are extending training with fellowships. Our data also indicate that 43% and 62% of respondents who graduated in 2009 and 2010, respectively, reported difficulties in finding staff positions compared to 11% of graduates from 2007 or 2008, suggesting slower graduate intake into the workforce.

Two years following graduation, nearly all respondents were successful in finding staff positions. Only 1 respondent who completed residency training in Canada and graduated before 2009 was seeking staff employment. The majority of Canadian residency graduates without a commitment of staff employment graduated in 2009 (6/23) or 2010 (16/23). An additional eight foreign MD graduates with Canadian fellowship training indicated no commitment for staff employment in Canada or elsewhere. All of these individuals were pursuing fellowship training, and by definition, employed during the survey sampling period.

Our survey found that 47% and 40% of 2009 and 2010 graduates, respectively, had staff employment offers upon graduation. In 2008, the number of domestic Canadian graduates/year rose above 30 for the first time since 1997 and peaked at 41 graduates in 2009, suggesting that graduate oversupply may be contributing to the difficulties of recent graduates in finding staff positions. Moreover, for reasons that are unclear, there were more graduates than expected over the allotted 25 entry-level training positions available per year, possibly due to resident transfer into the specialty or unforeseen lengthening of average training time due to maternity leave, other academic pursuits, or medical illness.

In our survey, 88% of licensed radiation oncologists were engaged in clinical research and 75% with Canadian residency training were practising in Canada. Approximately 9% were involved in basic science or laboratory-based research and a significant proportion of respondents (27%) had graduate degrees. Our results validate findings from two surveys of Canadian radiation oncology trainees performed in 2003 and 2009 that reported 77% and 92% of residents were interested in an academic career, and 80% and 78% planned to practice in Canada, respectively.^15^,^16^ Taken together, these data suggest a consistent and strong trend in graduate preference for academic practice in Canada.

Medical human resources and residency trainee numbers in Canada are largely a provincial responsibility with no co-ordinated national planning. Following the last national radiation oncology manpower crisis in the late 90s, CARO has routinely performed annual assessments of manpower, patient workloads, and equipment resources, and provides a multi-year trend report to CARO members at its annual scientific meeting. It tracks the number of graduates and fellows in Canada each year, and surveys department heads at Canadian radiation therapy centres to determine the number of available positions, anticipated retirements, and projected demand. CARO’s workforce assessment in 2009 marked a turning point that forecasted fewer job openings for the number of graduates looking for work,^8^ and provides evidentiary support of the findings from this study. In contrast, 10-year forward planning projections for the specialty based on patient utilization and radiation oncologist workload forecasting suggest that training program intake will likely need to be expanded at some point in the future.^17^ These current status and future projections for manpower highlight the need for effective links between training program administration and radiation oncology manpower planning.

CARO has taken a leadership role to help avoid the boom and bust cycles of radiation oncologist supply in Canada. In February 2011, CARO president Dr. Matthew Parliament announced temporary plans to voluntarily reduce the number of entry trainee positions by 10–15% and resist requests for transfer into radiation oncology programs from other disciplines as proactive measures to maintain some balance in the radiation oncology workforce and in response to recent and unforeseen market forces.^18^

### Relationship of Survey Data to National Specialty Manpower Planning

The survey findings in the context of the current CARO workforce projections raise a number of issues with respect to medical education within the specialty. We divide these into two general groups of issues, namely, implications for size of the annual training cohorts, and implications for the training curriculum.

Given that a change to resident training intake has a minimum 5-year lead time before its effect is seen on physician supply entering the workforce, co-ordination of intake should ideally be matched with valid and robust projections of workforce needs within a 5- to 10-year window. As demonstrated with previous workforce oversupply issues in Canada, the unanticipated training exodus of worried residents who left the specialty combined with a drop-off in medical student recruitment resulted in market overcorrection and a subsequent workforce shortage within three years as barriers for new entry-level radiation oncologist positions eased.

Although workforce monitoring by CARO is useful, planning is made complex by less predictable factors such as changes in retirement rates, recruitment initiatives to specific job descriptions, loss or entry of trainees during residency, level of government funding, and other economic issues. Given these uncertainties, we predict some variation in radiation oncology resident training intake will be required to avoid significant over- or under-training. Such responsiveness of training intake will require new paradigms for interactions between university deans of postgraduate training, program directors, national workforce planners, and advocates for funding of radiation oncologist positions. In addition, given our finding that a large number of domestic Canadian graduates undertook fellowships abroad and planned to return to Canada for staff employment, these individuals need to be included in workforce projections as current surveys do not capture this demographic.

With regard to the training curriculum itself, the survey results suggest potential enhancements to training objectives. These include increasing career counselling strategies within training programs (as well as improving communication of job opportunities more effectively across training programs), and targeting training opportunities and clinical experience in community practice such that residents have adequate exposure to consider a community-based career path early in their training.

## Conclusions

The majority of respondents to our survey were able to find staff employment in Canada. A sizeable proportion extended training with fellowships to be more competitive in the Canadian job market. Survey respondents agreed that new graduates may have more difficulty in finding Canadian radiation oncology staff positions in the near future. If Canadian graduates experience prolonged difficulties with workforce entry, physician migration rates to the United States and other countries are expected to rise. Regular assessment of trainee supply and employment of graduates along with ongoing assessment of future workforce demand based on cancer incidence projections, radiotherapy utilization, and standards of care^19^–^21^ will be required to avoid significant under- or over-training of radiation oncologists in Canada and to keep pace with the needs of Canadian cancer patients.

## Figures and Tables

**Table 1:** Demographic features of survey respondents and potential respondents.

Characteristic	Respondents* n = 148 No. (%)	Eligible respondents† *n* = 256 No. (%)	*p*-value
Gender			0.71
Male	95 (64.2)	169 (66.0)	
Female	53 (35.8)	87 (34.0)	
Radiation oncology residency training location			0.79
British Columbia	12 (8.1)	15 (5.9)	
Alberta	22 (14.9)	33 (12.9)	
Manitoba	5 (3.4)	6 (2.3)	
Ontario	51 (34.5)	85 (33.2)	
Quebec	35 (23.6)	77 (30.1)	
Nova Scotia	4 (2.7)	6 (2.3)	
Outside Canada	19 (12.8)	34 (13.3)	
Residency graduating year			0.37
2000	11 (7.4)	18 (7.0)	
2001	4 (2.7)	14 (5.5)	
2002	5 (3.4)	7 (2.7)	
2003	3 (2.0)	6 (2.3)	
2004	10 (6.8)	15 (5.9)	
2005	6 (4.2)	16 (5.9)	
2006	17 (11.5)	25 (9.8)	
2007	14 (9.5)	26 (10.2)	
2008	22 (14.9)	28 (10.9)	
2009	22 (14.9)	33 (12.9)	
2010	27 (17.6)	34 (13.3)	
Unknown	7 (4.7)	34 (13.3)	
Age (years) at completion of residency			N/A
≤29	31 (20.9)	N/A	
30–33	63 (42.6)	N/A	
34–37	40 (27.0)	N/A	
≥38	13 (8.8)	N/A	
Unknown	1 (0.7)	N/A	
Medical school			N/A
Canadian	116 (78.4)	N/A	
Foreign	32 (21.6)	N/A	
Highest educational degree besides MD			N/A
Bachelor’s	78 (52.7)	N/A	
Master’s	32 (21.6)	N/A	
Ph.D.	8 (5.4)	N/A	
None	30 (20.3)	N/A	
Other medical certification/licensing			N/A
Family medicine	7 (4.7)	N/A	
Internal medicine	3 (2.0)	N/A	
Radiation oncology-related	6 (4.1)	N/A	
MBBS	1 (0.7)	N/A	
None	123 (86.5)	N/A	
Unknown	8 (5.4)	N/A	

* data from survey

† demographic information of eligible respondents provided by Canadian training programs and radiation oncology personnel databases

*n* indicates numbers of respondents to each question

*Abbreviations*: MBBS = Bachelor of Medicine, Bachelor of Surgery

**Table 2:** Details of respondents’ fellowship training.

Feature	No. (%)
Fellowship training (*n* = 148)	
Yes	115 (77.7)
No	33 (22.3)
More than one fellowship (*n* = 103)	
Yes	15 (14.6)
No	88 (85.4)
Unknown	12 (N/A)
Length (months) of fellowship (*n* = 115)	
≤11	10 (8.7)
12	77 (67.0)
13–23	24 (20.9)
≥24	4 (3.5)
Fellowship location (*n* = 115, multiple locations permitted)	
Ontario	48 (38.7)
United States	29 (23.4)
Europe	14 (11.3)
British Columbia	11 (8.9)
Alberta	11 (8.9)
Australia	4 (3.2)
Manitoba	2 (1.6)
Quebec	2 (1.6)
United Kingdom	1 (0.9)
New Zealand	1 (0.9)
Japan	1 (0.9)
Fellowship focus (*n* = 115, multiple selections permitted)	
Modern radiotherapy delivery technology	73 (63.5)
Specific cancer site	47 (41.8)
Stereotactic radiotherapy	38 (33.0)
Research	26 (22.6)
Brachytherapy	21 (18.3)
Palliative care	5 (4.3)
Medical education	3 (2.6)
Proton and/or charged particle radiotherapy	2 (1.7)
Type of fellowship (*n* = 115; multiple selections permitted)	
Clinical only	4 (3.5)
Mostly clinical, but some research	64 (55.7)
Mostly research, but some clinical	35 (30.4)
Research leading to Master’s or Ph.D.	13 (11.3)

*n* indicates numbers of respondents to each question

*Radiotherapy delivery technology* includes 3D conformal radiotherapy, intensity-modulated radiotherapy, helical tomotherapy, or volumetric modulated arc therapy

*Stereotactic radiotherapy* includes stereotactic radiosurgery or stereotactic body radiotherapy

*Research* includes clinical, imaging, population health, basic or translational research

**Table 3:** Employment status and location of respondents.

	Staff position in Canada No. (%)	Staff position outside Canada No. (%)	Without a staff position No. (%)	*p*-value
All respondents (*n* = 148)	101 (68.2)	16 (10.8)	31 (21.0)	
Gender				0.45
Male respondents (*n* = 94)	65 (69.1)	8 (8.5)	21 (22.3)	
Female respondents (*n* = 53)	35 (66.0)	8 (15.1)	10 (18.9)	
Origin of MD degree				<0.0001
Canadian MD (*n* = 115)	89 (77.3)	5 (4.3)	21 (18.6)	
Foreign MD (*n* = 32)	11 (34.4)	11 (34.4)	10 (31.3)	
Radiation oncology residency training				<0.0001
Within Canada (*n* =129)	96 (74.4)	10 (7.8)	23 (17.8)	
Outside Canada (*n* =19)	5 (26.3)	6 (31.6)	8 (42.1)	
Canadian-trained radiation oncologists				<0.0001
2000 – 2008 graduates (*n* = 86)	76 (88.3)	9 (10.5)	1 (1.2)	
2009 – 2010 graduates (*n* = 43)	20 (46.5)	1 (2.3)	22 (51.2)	

*n* indicates numbers of respondents with the indicated characteristics

*Abbreviations*: MD = medical doctor degree; RO = radiation oncology

*Staff position* includes permanent or temporary staff employment or a signed contract for a staff position

**Table 4:** Respondents’ employment history and characteristics.

	No. (%)
Employment secured prior to completion of residency (*n* = 148)	
Yes	69 (46.6)
No	79 (53.4)
Employment secured prior to completion of fellowship (*n* = 94)	
Yes	82 (87.2)
No	12 (12.8)
Unknown (i.e. currently in fellowship)	21 (N/A)
Change of location of employment at any time (*n* = 117)	
Yes	17 (14.5)
No	100 (85.5)
Experienced periods of unemployment (*n* = 117)	
Yes	4 (3.4)
No	113 (96.6)
Current employed FTE (*n* = 117)	
≤0.5 FTE	3 (2.6)
0.6	1 (0.9)
0.7	5 (4.3)
0.8	4 (3.4)
0.9	2 (1.7)
1.0	102 (87.2)
Using fellowship skills/clinical focus in current practice (*n* = 88)	
Yes	79 (89.8)
No	9 (10.2)
Unknown	6 (N/A)
Type of research activity (*n* = 117, multiple selections permitted)	
Clinical research	109 (93.2)
Image-related research	30 (25.6)
Medical physics-related research	22 (18.8)
Basic science or laboratory-based research	11 (9.4)
No research activities	7 (5.9)
Protected time for research (*n* = 114)	
Yes	56 (49.1)
No	58 (50.9)
Unknown	3 (N/A)
Funding to support research activity (*n* = 117)	
Yes	69 (59.0)
No	31 (26.5)
Unsure	17 (14.5)

*n* indicates numbers of respondents to each question*.*

*Abbreviations*: FTE = full-time equivalent.

*Protected time for research* refers to any part of the work week dedicated to research activity in place of clinical duties as part of the employment contract.

**Table 5:** Respondents’ opinions on employment-related topics.

	No. (%)
Easy access to info about Canadian employment opportunities (*n* = 143)	
Yes	37 (25.9)
No	106 (74.1)
Unknown	5 (N/A)
Intention to stay in Canada after training (*n* = 145)	
Yes	121 (83.4)
No	24 (16.6)
Unknown	3 (N/A)
Difficulties in finding employment in Canada (*n* = 135)	
Yes	36 (26.7)
No	99 (73.3)
Unknown	13 (N/A)
Factors affecting decision to leave Canada (*n* = 12)	
Family considerations	4 (33.3)
Higher earning potential	3 (25.0)
Lack of job opportunities in Canada	2 (16.7)
Preference for “clinical only” practice	2 (16.7)
Lack of clinical-scientist opportunities	1 (8.3)
Unknown	4 (N/A)
Employment preferences (*n* = 144, multiple selections permitted)	
Small regional cancer centre (≤ 5 staff positions)	16 (11.1)
Medium-sided regional cancer center (>6 staff positions)	37 (25.7)
Medium-sided academic cancer center (>6 staff positions)	78 (54.2)
Large, urban-based academic cancer centre	83 (57.6)
Unknown	4 (N/A)

*n* indicates number of respondents to each question
